# Reconstructive trends and complications following parotidectomy: incidence and predictors in 11,057 cases

**DOI:** 10.1186/s40463-019-0387-y

**Published:** 2019-11-19

**Authors:** Cory Donovan Bovenzi, Peter Ciolek, Meghan Crippen, Joseph M. Curry, Howard Krein, Ryan Heffelfinger

**Affiliations:** Thomas Jefferson University Hospital Department of Otolaryngology- Head and Neck Surgery, 925 Chestnut Street, 6th Floor, Philadelphia, PA ,19107 USA

**Keywords:** Parotidectomy, Complications, Facial nerve, NSQIP, Reconstruction, Epidemiology, Readmission, Contour, Comorbidities, Infection

## Abstract

**Background:**

Parotidectomy is a common treatment option for parotid neoplasms and the complications associated with this procedure can cause significant morbidity. Reconstruction following parotidectomy is utilized to address contour deformity and facial nerve paralysis. This study aims to demonstrate national trends in parotidectomy patients and identify factors associated with adverse postoperative outcomes. This study includes the largest patient database to date in determining epidemiologic trends, reconstructive trends, and prevalence of adverse events following parotidectomy.

**Methods:**

A retrospective review was performed for parotidectomies included in the ACS-NSQIP database between January 2012 and December 2017. CPT codes were used to identify the primary and secondary procedures performed. Univariate and multivariate analysis was utilized to determine associations between pre- and perioperative variables with patient outcomes. Preoperative demographics, surgical indications, and common medical comorbidities were collected. CPT codes were used to identify patients who underwent parotidectomy with or without reconstruction. These pre- and perioperative characteristics were compared with 30-day surgical complications, medical complications, reoperation, and readmission using uni- and multivariate analyses to determine predictors of adverse events.

**Results:**

There were 11,057 patients who underwent parotidectomy. Postoperative complications within 30 days were uncommon (1.7% medical, 3.8% surgical), with the majority of these being surgical site infection (2.7%). Free flap reconstruction, COPD, bleeding disorders, smoking, and presence of malignant tumor were the strongest independent predictors of surgical site infection. Readmission and reoperation were uncommon at an incidence of 2.1% each. The strongest factors predictive of readmission were malignant tumor and corticosteroid usage. The strongest factors predictive of reoperation were free flap reconstruction, malignant tumor, bleeding disorder, and disseminated cancer. Surgical volume/contour reconstruction was relatively uncommon (18%). Facial nerve sacrifice was uncommon (3.7%) and, of these cases, only 25.5% underwent facial nerve reinnervation and 24.0% underwent facial reanimation.

**Conclusions:**

There are overall low rates of complications, readmissions, and reoperations following parotidectomy. However, certain factors are predictive of adverse postoperative events and this data may serve to guide management and counseling of patients undergoing parotidectomy. Concurrent reconstructive procedures are not commonly reported which may be due to underutilization or underreporting.

## Background

Parotid neoplasms are rare entities which comprise less than 3% of head and neck tumors. Management is primarily surgical, and depending on the extent and severity of disease, ranges from a superficial parotidectomy to a radical parotidectomy with facial nerve sacrifice [1]. There is ample data pertaining to site specific morbidity following parotidectomy (e.g. facial nerve weakness, salivary fistula, and Frey’s syndrome); however, the literature on general postoperative surgical morbidity following parotidectomy is lacking [2].

In the era of quality metrics, there is an ever-growing focus on improving surgical and postsurgical care, while reducing complications and cost. In 2001, The American College of Surgeons (ACS) piloted the first iteration of the National Surgical Quality Improvement Program (NSQIP) in the private hospital sector. The NSQIP database collects over 130 patient variable including preoperative risk factors, intraoperative variables, and postoperative complications. This data can be used by hospitals to track, analyze, and compare the quality of surgical care in a risk-adjusted manner. Currently, over 700 hospitals including 8 of the top 10 hospitals as ranked by the US News & world report participate in the program [3, 4].

Currently, the majority of literature pertaining to the outcomes and trends in parotidectomy are limited to single institution or small multi-institutional studies. Our investigation aims to provide a reference for national trends in parotidectomy surgery including patient demographics, pre-operative comorbidities, operative variables, and postoperative outcomes.

## Methods

### Data and study cohort

This is a retrospective analysis of the parotidectomy cases using the NSQIP participant user files from January 2012 through December 2017. The database was queried for all cases with the following *Current Procedural Terminology* (CPT) codes: 42410, 42,415, 42,420, 42,425, and 42,426. The resultant cohort was characterized by rates of patient characteristics, comorbidities, operative characteristics, and 30-day postoperative complications.

### Patient characteristics

Patient demographics examined included age, sex, and race. The indication for parotidectomy was elicited using the International Classification of Disease (ICD) version 9 or 10 code given for postoperative diagnosis and grouped into five major categories: malignant tumor, benign tumor, tumor not otherwise specified (NOS), other disease of parotid, and unclassified (Additional file 1 Table S1). Comorbidities defined by NSQIP include smoking (within the year prior to admission), weight loss (> 10% body weight in the 6 months prior to surgery), hypertension (requiring medication), dyspnea (on exertion or at rest), corticosteroid use (< 30 days prior to surgery), surgical site infection, non-independent functional status, and a number of additional preexisting medical conditions [5]. Obesity was defined as BMI (body mass index) > 30, derived from height and weight variables.

### Operative characteristics

Cases were further analyzed for operative characteristics. Variables including inpatient versus outpatient status, surgical specialty performing the parotidectomy (otolaryngology- head and neck surgery, general surgery, plastic surgery, and other), and total operative time were directly defined by NSQIP [5]. The principal parotidectomy CPT code was used to determine procedure extent (total vs. superficial) and management of the facial nerve (not dissected, dissected and preserved, sacrificed, and unknown). Additionally using CPT codes, rates of concurrent procedures were examined such as neck dissection, nerve monitoring, free flap, other volume restoration, reinnervation, and reanimation. Procedure type and associated CPT codes are given in Additional file 1 Table S1.

### Outcome variables

Operative outcomes were assessed including rates of 30-day surgical complications, medical complications, reoperation, readmission, and length of hospital stay. Specific surgical complications include superficial surgical site infection (SSI), deep SSI (defined as “deep” or “organ space” SSI), wound dehiscence, and hemorrhage/hematoma formation. Specific medical complications include cardiac arrest, myocardial infarction, stroke, ventilator requirement > 48 h, pneumonia, and various others enumerated in Table 3.

All complication variables were coded by NSQIP [5] with the exception of hemorrhage/hematoma, which was derived from the relevant ICD-9 and 10 codes given for readmission or reoperation diagnosis (Additional file 1 Table S1). The occurrence of this complication therefore refers to cases requiring reoperation or readmission for postoperative hemorrhage or hematoma formation within 30 days of the principal procedure.

### Statistical analysis

Descriptive statistics were used to summarize by rates of patient characteristics, comorbidities, operative characteristics, and 30-day postoperative complications across the study cohort. In order to identify the patient and procedure characteristics associated with an increased risk for postoperative morbidity, binary logistic regression was performed. A regression model was generated to account for patient demographics, comorbidities, procedure extent, indication, setting, surgical specialty, and concurrent procedures. All comorbidities occurring at a rate of ≥0.5% of the overall cohort were included. Cases with unknown values were excluded, leaving 8811 cases for analysis. Regression analysis was performed for each complication occurring at a rate of ≥0.5%, including wound dehiscence, surgical site infection, hemorrhage/hematoma, overall surgical and medical complications, readmission, and reoperation. All variables found to be significantly associated with each of these complications are listed in Table 4.

All statistical analyses were performed using SPSS (Statistical Package for the Social Sciences), version 23 (IBM, Armok, NY). *P*-values of < 0.05 were considered to be statistically significant. This analysis was determined to be exempt from Institutional Review Board approval due to the de-identified nature of the dataset.

## Results

A total of 11,057 patients were identified as having undergoing parotidectomy between January 1, 2012 and December 31, 2017. Of these patients, the majority were male (53.0%), white (70.4%), and ages 61–70 (26.6%). In this cohort, parotidectomy was most commonly performed for benign neoplasms (45.2%), followed by malignant neoplasms (29.7%) and tumors of uncertain significance (13.0%). Parotidectomy was indicated for non-neoplastic disease (e.g. chronic parotitis) in 8.8% of cases. Examination of various patient comorbidities found hypertension (45.4%), obesity (39.7%), and smoking (23.5%) to be most common (Table 1). Patients undergoing parotidectomy for benign lesions were significantly more likely to be smokers compared to those undergoing parotidectomy for malignant lesions (30.5% vs. 13.9%, *p* < 0.001). This may represent a preponderance of Warthin’s tumors in smokers.

**Table 1:** Demographics and Patient Characteristics

	N	%
Age		
< 50	2772	25.3
51–60	2389	21.6
61–70	2920	26.6
71–80	1977	18.0
> 80	902	8.2
Sex		
Male	5862	53.0
Female	5195	47.0
Race		
White	7784	70.4
Black	713	6.4
Other	703	6.4
Unknown	1857	16.8
Surgical Indication		
Malignant tumor	3288	29.7
Benign tumor	4995	45.2
Tumor Not Otherwise Specified	1433	13.0
Other disease of parotid	977	8.8
Other/unknown	364	3.3
Comorbidities		
Bleeding disorder	248	2.2
Diabetes	1707	15.4
On dialysis	39	0.4
Disseminated cancer	364	3.3
Dyspnea	536	4.8
Dependent functional status	129	1.2
Congestive Heart Failure	29	0.3
Chronic Obstructive Pulmonary Disease	518	4.7
Hypertension	5019	45.4
Obesity (BMI > 30)	3857	39.7
Smoker	2602	23.5
Corticosteroids	363	3.3
Wound infection	155	1.4
Weight loss	68	0.6
ASA class		
1 (No disturbance)	990	9.0
2 (Mild disturbance)	5355	48.5
3 (Severe disturbance)	4395	39.8
4 (Life threatening)	307	2.8

In this cohort, superficial parotidectomy was performed 2.4 times more frequently than total parotidectomy. Concurrent neck dissection was performed in 19.9% of cases. Reconstruction involving volume restoration was performed in 12.4% of cases, including free flaps (3.6%), local flaps (5.4%), fat/dermal grafts (2.0%), and allografts (1.8%) (Table 2). Total parotidectomy cases were significantly more likely to involve a concurrent volume restoration procedure compared to superficial parotidectomy (17.8% vs. 10.3%, *p* < 0.001). Facial nerve sacrifice was recorded in 3.7% of cases (Table 2). In this subset of patients, 25.5% underwent a concurrent reinnervation procedure, and 24.0% underwent a concurrent reanimation procedure (Table 2).

**Table 2:** Operative Characteristics

Non-Reconstructive Operative Characteristics		
	N	%
Parotidectomy extent		
Total	3229	29.2
Superficial	7828	70.8
Management of facial nerve		
Not dissected	1683	15.2
Dissected and preserved	8575	77.6
Sacrificed	405	3.7
Unknown	394	3.6
Concurrent procedures		
Neck dissection	2204	19.9
Nerve monitoring	263	2.4
Surgeon specialty		
Otolaryngology	10,094	91.3
General Surgery	745	6.7
Plastic Surgery	180	1.6
Other	38	0.3
Setting		
Inpatient	4130	37.4
Outpatient	6927	62.6
Operative time, mean [SD] (min)	185.3 [141.9]	
Utilization of Reconstructive Procedures		
	N	%
Free flap	393	3.6
Other volume restoration	998	9.0
Reinnervation	205	1.9
Reanimation	124	1.1

Overall, 5.1% of patients experienced a postoperative complication within 30 days of the procedure. The majority were surgical complications, occurring in 3.8% of patients. Surgical site infection occurred most commonly, with 1.9% developing a superficial SSI, and 0.8% of patients developing a deep SSI. Unplanned reoperation was observed in 2.1% of cases. Medical complications occurred in 1.7% of patients, the most common of these being pneumonia (0.4%) and urinary tract infection (0.4%). The mean length of stay was 1.65 days with an unplanned 30-day readmission rate of 2.1% (Table 3). On binary logistic regression, various patient and procedure related characteristics were found to be independently associated with an increased risk of medical and surgical complications. Of the demographic variables assessed, increasing age was found to be significantly associated with increasing risk for overall medical complications but was not associated with surgical complications (Additional file 2 Table S2). Relative to those with a benign neoplasm, patients undergoing parotidectomy for a malignant neoplasm were found to be at significantly higher risk for overall surgical and medical complications as well as reoperation and readmission (Additional file 2 Table S1). Both smoking and COPD (chronic obstructive pulmonary disease) were independently predictive of wound dehiscence and surgical site infection. Of the concurrent procedures examined, only volume restoration procedures were predictive of surgical complications. Free flap procedures were independently associated with the highest risk for overall surgical complications with an odds ratio of 2.87 (Table 4). Neck dissection, reanimation, and reinnervation procedures were not found to significantly increase risk for any measure of postoperative morbidity (Table 5).

**Table 3:** Complication Rates Following Parotidectomy

	N	%
Medical Complications, Overall	186	1.7
Cardiac arrest	13	0.1
Myocardial Infarction	22	0.2
Stroke	24	0.2
Reintubation	35	0.3
Ventilator-dependent > 48 h	27	0.2
Sepsis/septic shock	33	0.3
Pneumonia	49	0.4
Pulmonary Embolism	13	0.1
Acute Kidney Injury	14	0.1
Urinary Tract Infection	43	0.4
Deep Vein Thrombosis	20	0.2
Surgical Complications, Overall	418	3.8
Wound disruption	50	0.5
Superficial SSI	209	1.9
Deep SSI	84	0.8
Hemorrhage/Hematoma	100	0.9
Unplanned Readmission	236	2.1
Unplanned Reoperation	234	2.1
Length of Stay, mean [SD] (days)	1.65 [4.00]

**Table 4:** Multivariable Logistic Regression of Adverse Events Following Parotidectomy, Significant Variables

	OR	95% CI	p
Surgical site infection			
Male	1.41	1.01–1.96	0.042
Bleeding disorder	2.21	1.20–4.07	0.011
Diabetes	1.66	1.17–2.37	0.005
COPD	2.08	1.25–3.45	0.005
Hypertension	1.53	1.08–2.16	0.017
Smoking	1.85	1.32–2.59	< 0.001
Malignant tumor	1.70	1.09–2.67	0.021
Free flap reconstruction	2.87	1.67–4.93	< 0.001
Other volume restoration	1.66	1.07–2.57	0.025
Wound Dehiscence			
COPD	2.65	1.03–6.87	0.044
Smoking	2.58	1.23–4.34	0.013
Malignant tumor	4.33	1.36–13.77	0.013
Other disease of parotid	5.31	1.51–18.71	0.009
Free flap reconstruction	3.25	1.39–7.58	0.006
Other volume restoration	3.39	1.60–7.19	0.001
Hematoma/seroma			
Free flap reconstruction	3.13	1.43–6.83	0.004
Readmission			
Disseminated cancer	2.02	1.30–3.15	0.002
Hypertension	1.44	1.05–1.97	0.023
Corticosteroids	2.62	1.69–4.07	< 0.001
Wound infection	2.07	1.10–3.90	0.025
Malignant tumor	3.00	1.94–4.65	< 0.001
Other disease of parotid	2.21	1.31–3.75	0.003
Reoperation			
Bleeding disorder	1.82	1.02–3.26	0.044
Disseminated cancer	1.66	1.01–2.71	0.044
Malignant tumor	2.64	1.65–4.20	< 0.001
Free flap reconstruction	3.56	2.30–5.50	< 0.001

OR = odds ratio, CI = confidence interval.

**Table 5:** Multivariable Logistic Regression of Adverse Events Following Parotidectomy: Odds Ratios Associated with Reconstructive Procedures

	OR	95% CI	p
Surgical site infection			
Free flap reconstruction	2.87	1.67–4.93	< 0.001
Other volume restoration	1.66	1.07–2.57	0.025
Reinnervation	0.93	0.42–2.22	0.961
Reanimation	0.94	0.35–2.62	0.959
Wound Dehiscence			
Free flap reconstruction	3.25	1.39–7.58	0.006
Other volume restoration	3.39	1.60–7.19	0.001
Reinnervation	0.47	0.12–2.66	0.563
Reanimation	1.90	0.50–7.25	0.350
Hemorrhage/Hematoma			
Free flap reconstruction	3.13	1.43–6.83	0.004
Other volume restoration	1.02	0.46–2.27	0.962
Reinnervation	0.42	0.12–2.46	0.537
Reanimation	2.11	0.63–7.05	0.227
Readmission			
Free flap reconstruction	1.29	0.72–2.32	0.399
Other volume restoration	1.24	0.79–1.97	0.353
Reinnervation	0.72	0.28–1.86	0.493
Reanimation	1.18	0.44–3.18	0.749
Reoperation			
Free flap reconstruction	3.56	2.30–5.50	< 0.001
Other volume restoration	1.50	0.95–2.38	0.083
Reinnervation	1.03	0.51–2.07	0.937
Reanimation	1.30	0.62–2.72	0.481

OR = odds ratio, CI = confidence interval.

SSI = surgical site infection, SD = standard deviation.

## Discussion

To date, this is the most robust dataset to evaluate national trends in parotidectomy surgery. The post-parotidectomy complication rates seen here are the most substantial information available to counsel patients with, and are the most thorough and recent data available thus far. There has been a review of the NSQIP data from 2006 to 2011 which evaluated 2919 patients which also reported low rates of medical and surgical complications [6]. In addition to evaluating a newer and larger dataset, the current study is likely to be of increased validity, as NSQIP removed several unreliable outcomes variables from the dataset in 2012 [5]. The data here correspond to existing literature that post-parotidectomy medical and surgical complications increase with increasing age [7, 8]. However, the rate of surgical site complications seen here are lower than that of those reported in some single-center studies [9], which highlights the institutional differences in complication rates, and the national average estimated with this study may become an important standard for quality of care assessments at an institutional level. The difference in complication rates is an important feature for further investigation as comparison of quality of care becomes increasingly important. While one may assume that high volume centers would have lower complication rates, in parotidectomy for benign disease, a previous study has shown that surgeon experience did not seem to be associated with complication rates- however, at that institution they admit that more complicated cases were reserved for more experienced surgeons [10].

There is a significant body of research available evaluating single-site experiences with common complications after parotidectomy. These studies are mostly limited to facial paresis, Frey syndrome, hypoesthesia, and contour deformity. Unfortunately, this dataset did not capture these complications. However, there is evidence that parotidectomy reconstructive techniques may mitigate some of these adverse outcomes.

Facial paresis following parotidectomy is a common finding that is typically self-limiting and limited to marginal mandibular nerve weakness [11]. This risk may be mitigated by performing a limited surgical approach [12–14], or if there is reconstruction with a muscular flap, with or without an abdominal fat graft [15, 16]. Frey syndrome is a historically common complication following parotidectomy described as gustatory sweating due to aberrant reinnervation of secretory parasympathetic fibers from parotid tissue to dermal tissue. Reconstructive procedures that provide a barrier between parotid tissue and the dermal surface have been shown to decrease the incidence of Frey Syndrome [15, 17–20]. These reconstructions include rotating a sternocleidomastoid flap into the defect, elevating a superficial musculoaponeurotic flap prior to performing parotidectomy, grafting free abdominal fat, and insertion of acellular dermis. There is continued debate on the most effective reconstruction method. A meta-analysis on the subject suggests that all of the above interventions decrease Frey syndrome but acellular dermis implants may be associated with higher infection rates [14].

These reconstruction options also help improve postoperative facial symmetry, especially in cases of total parotidectomy. Parotidectomy, as a common procedure performed in a highly cosmetic region is uniquely poised to benefit from advances in maintaining symmetric volume and minimizing visible scarring. Adequate exposure and improved scar cosmesis has been shown to follow from the trend of utilizing a facelift incision in parotidectomy [21, 22]. Following large parotidectomy defects encountered in total parotidectomy and radical parotidectomy, muscular flaps and fat grafting have shown to reduce contour asymmetry [16, 22, 23]. Additionally, another level of complexity occurs when parotidectomy requires concurrent skin and/ or facial nerve sacrifice which typically is necessary in cases of malignant lesions with adjacent tumor invasion. Fortunately, multiple reconstructive options exist for these defects- for skin and soft tissue reconstruction, anterolateral thigh free flaps have been the mainstay of treatment due to the large vessel caliber available for microvascular anastomosis and variable size and shape available with this option [24–27]. Additionally, if the patient requires postoperative radiation, the volume of this free flap after radical parotidectomy reconstruction has been shown to be reduced by only 8% [28]. Other options available for free flap reconstruction include latissimus dorsi free flap and superficial inferior epigastric artery free flap [29]. However, the latissimus dorsi free flap is more difficult to harvest concurrently and is likely to result in higher postoperative donor site morbidity. The superficial inferior epigastric artery free flap has much smaller caliber vessels which may increase risk of flap failure and decrease ability for postoperative flap monitoring. For cases with facial nerve sacrifice, it is surprising that only 25.5% of patients have documented reinnervation procedures, and only 24% have documented reanimation procedures (Table 2). This low rate of facial reinnervation and reanimation following radical parotidectomy is also underscored in a recent review of NSQIP [30]. This leads to the question of whether these procedures fail to be coded and thus detected by the database or whether these procedures are truly not performed commonly in cases with facial nerve sacrifice. There is encouraging data that would encourage immediate reinnervation and reanimation in these cases [31].

Surgical site infection following parotidectomy is not a well-researched complication, likely due to its low incidence. Our study showed an incidence of 2.7%, with the highest predictors of SSI being concurrent free flap and pre-existing bleeding disorder. This would provide evidence that the mechanism for post-parotidectomy wound infections is from hematoma formation that becomes subsequently infected. Following this logic, as post-parotidectomy hematomas are more common in males due to rich blood supply of hair follicles in the overlying dermis, it is not surprising that males were also found to have significantly increased risk for developing SSI compared to females. In fact, in the literature, there been an association with drain output greater than 50 ml in 24 h to be predictive of surgical site infection [32, 33]. Since abdominal fat grafts have also been shown to be associated with increased surgical site drainage [20] and larger defects are more likely to prompt contour adjustments, it is concordant that volume restoration was associated with a higher rate of SSIs in our dataset (Table 4).

Poor wound-healing may also contribute to SSI and wound dehiscence and this process may be exacerbated by the systemic factors seen to increase risk for both of these outcomes in this study. These factors include comorbid COPD, diabetes, and a smoking history. These results contradict a recent single-center study which did not find an association with diabetic status and post-parotidectomy surgical site infection [34]. However, they are consistent with previous data on smoking increasing surgical complication risk following parotidectomy [6]. Many institutions, including our own, preoperatively counsel patients on the importance of smoking cessation prior to parotidectomy and this study provides substantiated data to help further justify and aid this counseling process.

Medical complications that do not involve the surgical site are not well-studied and the data provided here point towards a relatively low incidence of these adverse outcomes. These complications appear to be associated with advanced age, malignancy, weight loss, wound infection, concomitant corticosteroid use, free flap reconstruction, and undergoing surgery in an inpatient setting (Additional file 2 Table S2). These associated factors are unsurprising as older patients and those who have malignancy or require free flap reconstruction would be expected to be at an elevated risk preoperatively and those with a higher ASA (American Society of Anesthesiologists) physical status level would be more likely to have their surgery performed at a hospital-based setting.

### Limitations

This study is subject to several limitations inherent to the use of a large national database. The most notable of these is the absence of procedure-specific outcomes such as facial nerve paresis and the inability to assess complications beyond the 30-day postoperative period. There is also the issue of procedures that are not coded correctly. For example, in this dataset, facial nerve monitoring was only coded in 2.4% of cases, despite this being a routine aspect of parotidectomy and is considered by many surgeons to be the standard of care. The small incidence of facial nerve monitoring in this study is likely due to lack of proper documentation and exemplifies a fundamental limitation of using this retrospective dataset. Further, the possibility of confounding cannot be excluded given the absence of disease-specific variables such as tumor stage. However, the data captured by NSQIP has been shown to be of high validity, particularly when compared to comparable population datasets derived from administrative claims data [35]. The method of data collection is worth noting, as NSQIP is unique in utilizing trained clinical reviewers to extract data from the medical record. As a result, NSQIP data has been shown to capture 61% more complications than comparable population datasets derived from administrative claims data [35]. However, this method of data collection may in part explain the lower than expected rate of concurrent procedures such as nerve monitoring, as the associated CPT code may not have been clearly documented in the medical record. While the strength of this analysis lies in the statistical power afforded by the NSQIP database, these results should be interpreted with these limitations in mind.

## Conclusions

As the US health care system moves towards a quality-based outcome model, the information available in this data set regarding readmission and reoperation rates are of importance for risk-stratification of these patients. Not only surgeons, but hospital administrators will be interested in readmission rates for this relatively safe procedure. Thus, being aware of these variables (Table 4) may help with patient selection for inpatient versus outpatient surgery as well as frequency and duration of follow up for patients with these identified risk factors. Further study is necessary to determine the cause of readmission and reoperation for these patients and whether these readmissions are avoidable.

## Supplementary information

**Additional file 1:** **Table S1.** Descriptive Variables Derived from Billing Codes (DOCX 16 kb)

**Additional file 2:** **Table S2.** Multivariable Logistic Regression, Overall Complications (DOCX 29 kb)

## Data Availability

The NSQIP dataset used in this study are available via the American College of Surgeons website: https://www.facs.org/quality-programs/acs-nsqip/participant-use/puf-form.

## Abbreviations

ACS: American College of Surgeons
ASA: American Society of Anesthesiologists
BMI: body mass index
COPD: chronic obstructive pulmonary disease
CPT: Current Procedural Terminology
ICD: International Classification of Disease
NOS: not otherwise specified
NSQIP: National Surgical Quality Improvement Program
SPSS: Statistical Package for the Social Sciences
SSI: surgical site infection
