# Supplementary material for: Reconstructive trends and complications following parotidectomy: incidence and predictors in 11,057 cases
Source: J Otolaryngol Head Neck Surg. 2019 Nov 19;48:64. doi: 10.1186/s40463-019-0387-y (PMC6862743; doi:10.1186/s40463-019-0387-y)
Supplement: Supplementary file 1 — Additional file 1: Table S1. Descriptive Variables Derived from Billing Codes (DOCX 16 kb) [file 40463_2019_387_MOESM1_ESM.docx]

Table S1 Descriptive Variables Derived from Billing Codes

|  | ICD-9 Codes | ICD-10 Codes |
| --- | --- | --- |
| **Indication** | | |
| Malignant tumor | 140–209 | C00-C96 |
| Benign tumor | 210–229 | D10-D36 |
| Tumor not otherwise specified | 235–239 | D37-D49 |
| Other disease of parotid | 527 | K11 |
| **Complications** | | |
| Hemorrhage/hematoma | 998.1 | L76.2, L76.3 |
|  | CPT Codes | |
| **Procedure Extent** | | |
| Superficial | 42,410, 42,415 | |
| Total | 42,420, 42,425, 42,426 | |
| **Facial Nerve Management** | | |
| Not dissected | 42,410 | |
| Dissected and preserved | 42,415, 42,420 | |
| Sacrificed | 42,425 | |
| Unknown | 42,426 | |
| **Concurrent Procedure** | | |
| Neck dissection | 38,724, 38,542, 38,700, 42,426 | |
| Nerve monitoring | 95,867, 95,940, 95,920 | |
| Volume restoration | | |
| Free flap | 15,756, 15,757, 15,758, 20,968 | |
| Local Flap | 15,732, 15,733 | |
| Fat graft | 15,770, 20,926 | |
| Allograft | 15,275, 15,777 | |
| Reinnervation | 64,716, 64,864, 64,868, 64,885, 64,886, 64,910, 64,727 | |
| Reanimation | 15,840, 15,841, 15,845, 67,917, 67,900 | |
| ICD = International Classification of Disease, CPT = Current Procedural Terminology | | |
