# Supplementary material for: Reconstructive trends and complications following parotidectomy: incidence and predictors in 11,057 cases
Source: J Otolaryngol Head Neck Surg. 2019 Nov 19;48:64. doi: 10.1186/s40463-019-0387-y (PMC6862743; doi:10.1186/s40463-019-0387-y)
Supplement: Supplementary file 2 — Additional file 2: Table S2. Multivariable Logistic Regression, Overall Complications (DOCX 29 kb) [file 40463_2019_387_MOESM2_ESM.docx]

Table S2 Multivariable Logistic Regression, Overall Complications

|  | | Surgical Complications  OR (95% CI) | Medical Complications  OR (95% CI) |
| --- | --- | --- | --- |
| Age | < 50 | 1.0 (Ref) | 1.0 (Ref) |
|  | 51–60 | 1.15 (0.77–1.71) | 1.22 (0.53–2.81) |
|  | 61–70 | 0.92 (0.62–1.37) | 2.34 (1.13–4.85)* |
|  | 71–80 | 0.79 (0.51–1.23) | 2.30 (1.08–4.90)* |
|  | > 80 | 0.87 (0.52–1.44) | 4.63 (2.14–10.01)*** |
| Sex | Female | 1.0 (Ref) | 1.0 (Ref) |
|  | Male | 1.28 (0.98–1.67) | 1.21 (0.81–1.80) |
| Race | White | 1.0 (Ref) | 1.0 (Ref) |
|  | Black | 0.87 (0.53–1.44) | 0.97 (0.44–2.18) |
|  | Other | 0.83 (0.48–1.43) | 0.90 (0.38–2.10) |
| Indication | Benign tumor | 1.0 (Ref) | 1.0 (Ref) |
|  | Malignant tumor | 1.66 (1.14–2.41)** | 1.87 (1.07–3.26)* |
|  | Tumor not otherwise specified | 0.72 (0.43–1.20) | 0.96 (0.44–2.09) |
|  | Other disease of parotid | 1.46 (0.93–2.28) | 1.01 (0.41–2.48) |
| Comorbidities | Bleeding disorder | 1.74 (1.03–2.95)* | 0.89 (0.40–1.98) |
|  | Diabetes | 1.34 (0.99–1.80) | 1.29 (0.86–1.94) |
|  | Disseminated cancer | 1.43 (0.90–2.28) | 1.87 (1.13–3.09)* |
|  | Dyspnea | 1.14 (0.71–1.83) | 1.39 (0.75–2.56) |
|  | Dependent functional status | 1.36 (0.60–3.08) | 1.57 (0.66–3.72) |
|  | COPD | 1.79 (1.16–2.77)** | 1.32 (0.70–2.50) |
|  | Hypertension | 1.48 (1.12–1.96)** | 1.25 (0.84–1.86) |
|  | Smoking | 1.62 (1.21–2.15)*** | 0.90 (0.54–1.48) |
|  | Corticosteroids | 1.43 (0.87–2.34) | 1.86 (1.02–3.37)* |
|  | Wound infection | 1.45 (0.78–2.68) | 2.39 (1.26–4.53)** |
|  | Weight loss | 1.28 (0.48–3.43) | 3.17 (1.26–7.99)* |
| Procedure extent | Superficial | 1.0 (Ref) | 1.0 (Ref) |
|  | Total | 1.06 (0.81–1.39) | 0.89 (0.62–1.27) |
| Setting | Outpatient | 1.0 (Ref) | 1.0 (Ref) |
|  | Inpatient | 1.74 (1.29–2.33)*** | 3.21 (2.04–5.04)*** |
| Surgeon specialty | Otolaryngology | 1.0 (Ref) | 1.0 (Ref) |
|  | Other | 1.26 (0.87–1.83) | 1.41 (0.88–2.27) |
| Concurrent procedures | Neck dissection | 1.00 (0.71–1.41) | 0.81 (0.53–1.24) |
|  | Nerve monitoring | 0.66 (0.27–1.64) | 1.61 (0.64–4.08) |
|  | Free flap | 2.87 (1.87–4.38)*** | 2.89 (1.76–4.74)*** |
|  | Other volume restoration | 1.52 (1.05–2.20)* | 1.41 (0.86–2.14) |
|  | Reinnervation | 0.76 (0.37–1.56) | 1.52 (0.72–3.18) |
|  | Reanimation | 1.40 (0.68–2.86) | 0.34 (0.09–1.16) |
| * = *p* < 0.05, ** = *p* < 0.01, *** = p < 0.001 | | | |

OR = odds ratio, CI = confidence interval
